# Cases for Diagnosis

**Published:** 1908-07-04

**Authors:** 


### Cases for Diagnosis.

### A CASE OF ACUTE ABDOMINAL PAIN.

E. S., Æt. 24. A single woman employed as a tea-packer was admitted into hospital for acute abdominal pain.

There was nothing of importance to be obtained from the family history. Patient had always lived in London. Eight years previously to admission she had been in hospital for typhoid fever, from which disease she made a good recovery. For the past year she had been working in a tea warehouse; the work had necessitated much standing; the hours had been long; and she had been feeling increasingly weaker during the past four months, with occasional attacks of shortness of breath on exertion. For the past three weeks there had been slight œdema of the feet, and constipation, from which she had always suffered slightly, had been troublesome. The day before admission she came from work feeling "queer," was persuaded to have some eels for supper, and then went to bed. Having been in bed about three hours without sleeping, she was suddenly seized with acute abdominal pain and nausea, accompanied by a marked feeling of depression—"she thought that she was dying." Within a quarter of an hour she vomited, apparently stomach-contents, with a little bile-stained material later. The pain continued, she became very collapsed, cold, and pale, and in the course of another half-hour, after another attack of vomiting (without hæmatemesis), her friends decided to bring her to hospital.

On admission, the pulse-rate was 120; temperature, 96.2° F.; and respirations, 28 per minute. She was very anæmic, and in a whining voice complained of general abdominal tenderness, with acute pain at the umbilicus. Shortly after admission she again vomited material of the type described above. The face was characterised by an extremely "anxious" expression, the eyes bright, the mucosa very pale; the tongue was dry, with a little white fur on the dorsum; the pulse was noted to be a little irregular. On further examination, it was noticed that respiratory movements were entirely thoracic, the alæ nasi were working, the abdominal wall was rigid, and there was general cutaneous hyperæsthesia, most marked below the umbilicus. It was noted that the feet, ankles, and lower parts of the legs were slightly œdematous, and she complained of some pain in them. The liver dulness was normal, no fluid could be detected in the flanks. A rectal wash-out brought away a little fæcal matter; nothing was felt per rectum. There was no vaginal discharge; she thought that the last monthly period had been about three weeks before; for the past six mouths she had been a little irregular. No other abnormality could be detected on examination. The temperature was not raised. Immediate laparotomy was advised, but this the patient refused. Three hours after the onset of the pain her condition was distinctly worse—pulse, 128. The abdominal pain continued—general and not localised. A small quantity of urine passed contained a trace of albumin. Between 3 A.M. and 8 A.M. she was sick three times; the vomit was watery and slightly bile-stained. At 9 A.M. the œdema of the legs was extending; the bowels were opened, and some bright red blood was passed, together with mucus and fæcal matter. At midday—*i.e.*, twelve hours after the onset of symptoms—the urine contained a quantity of albumin, about 20 parts per 1,000, it was acid in reaction, slightly red in colour, and contained blood in fair quantity. The œdema was now spreading up to the groins, and she complained of much pain in the lower extremities. Her condition was grave during the day, the pulse remaining about 120 per minute. The bowels were opened three times towards the evening—blood-streaked slime was passed. The abdomen became somewhat distended, and hyper-resonant on percussion. She had a restless night, with a pulse varying between 120 and 130 per minute, but there was no further sickness. She was allowed ice to suck; saline infusions were given into the axillæ, and a saline given per rectum was retained.

Next morning the abdominal pain was less. She said that "she felt better." The pulse remained about 120. She was allowed water by the mouth. There was no more sickness, but the abdomen remained rigid. Milk feeds were started in small quantities in the evening, and were retained; general abdominal pain and pulse were better. From this time onwards she began to improve. The albumin, which on the second day after admission had been 5 parts per 1,000, further diminished to a trace. The œdema of the legs persisted. Six days after admission the œdema of both lower extremities was still marked, the trace of albumin in the urine continued, but no casts or blood could be detected. There was general abdominal tenderness, but no rigidity, the abdominal wall moving fairly well on respiration; no tumour or abdominal dullness could be found. Seventeen days after admission slight abdominal distension was still present and a trace of albumin in the urine as before; the œdema persisted; the bowels were inclined to be constipated—nothing else abnormal was to be noted. After five weeks in hospital she could sit up in bed. The veins of the anterior abdominal wall were visible and somewhat distended. There was no longer albuminuria, and the lower extremities were less œdematous. After ten weeks she was sitting up in a chair, and shortly afterwards was allowed to walk about. A blood-count made shortly after admission showed a chlorotic type of blood. She was discharged from hospital to a convalescent home twelve weeks after admission.

Seven months later she was readmitted for acute abdominal pain, collapse, and cyanosis. She was very anæmic, and there was œdema of the lower extremities. She had been feeling "poorly" a week prior to admission. She never rallied, and died within a few hours.

### SOLUTION TO CASE FOR DIAGNOSIS.

When first admitted the possibilities discussed were: (1) Gastro-enteritis; (2) lead colic (she worked with lead paper); (3) general peritonitis from a perforated gastric ulcer

The last of these was the diagnosis made. Twelve hours later, when the other signs appeared, the case was considered to be one of mesenteric and inferior vena caval thrombosis, involving the renal veins.

At the Autopsy Seven Months after Discharge.

The heart was pale, showing evidences of fibroid change. The inferior vena cava was thickened and shrunken from its formation as far up as the hepatic tributaries, the wall in places being of an almost calcareous hardness. There was old and recent ante-mortem thrombosis almost obliterating the lumen; the renal veins showed similar, though less marked change. About two feet of ileum showed evidences of old peritonitis with thickening of the mesentery. No other abnormalities were found, with the exception of some œdema of the lungs about their bases. The kidneys were larger than normal, and somewhat tough.

